# Fatty acids characterization, oxidative perspectives and consumer acceptability of oil extracted from pre-treated chia (*Salvia hispanica* L.) seeds

**DOI:** 10.1186/s12944-016-0329-x

**Published:** 2016-09-20

**Authors:** Muhammad Imran, Muhammad Nadeem, Muhammad Faisal Manzoor, Amna Javed, Zafar Ali, Muhammad Nadeem Akhtar, Muhammad Ali, Yasir Hussain

**Affiliations:** 1 Institute of Home and Food Sciences, Faculty of Science and Technology, Government College University, Faisalabad, Pakistan; 2 Department of Dairy Technology, University of Veterinary and Animal Sciences, Lahore, Pakistan; 3 Department of Food Science, Nutrition and Home Economics, Government College University, Faisalabad, Pakistan; 4 Department of Food Science and Nutrition, Government College University, Sub Campus Layyah, Pakistan

**Keywords:** *Salvia hispanica*, Size reduction, Thermal processing, Physical parameters, Oxidation, Lipid profile, Storage

## Abstract

**Background:**

Chia (*Salvia hispanica* L.) seeds have been described as a good source of lipids, protein, dietary fiber, polyphenolic compounds and omega-3 polyunsaturated fatty acids. The consumption of chia seed oil helps to improve biological markers related to metabolic syndrome diseases. The oil yield and fatty acids composition of chia oil is affected by several factors such as pre-treatment method and size reduction practices. Therefore, the main mandate of present investigate was to study the effect of different seed pre-treatments on yield, fatty acids composition and sensory acceptability of chia oil at different storage intervals and conditions.

**Methods:**

Raw chia seeds were characterized for proximate composition. Raw chia seeds after milling were passed through sieves to obtain different particle size fractions (coarse, seed particle size ≥ 10 mm; medium, seed particle size ≥ 5 mm; fine, seed particle size ≤ 5 mm). Heat pre-treatment of chia seeds included the water boiling (100 C°, 5 min), microwave roasting (900 W, 2450 MHz, 2.5 min), oven drying (105 ± 5 °C, 1 h) and autoclaving (121 °C, 15 lbs, 15 min) process. Extracted oil from pre-treated chia seeds were stored in Tin cans at 25 ± 2 °C and 4 ± 1 °C for 60–days and examined for physical (color, melting point, refractive index), oxidative (iodine value, peroxide value, free fatty acids), fatty acids (palmitic, stearic, oleic, linoleic, α-linolenic) composition and sensory (appearance, flavor, overall acceptability) parameters, respectively.

**Results:**

The proximal composition of chia seeds consisted of 6.16 ± 0.24 % moisture, 34.84 ± 0.62 % oil, 18.21 ± 0.45 % protein, 4.16 ± 0.37 % ash, 23.12 ± 0.29 % fiber, and 14.18 ± 0.23 % nitrogen contents. The oil yield as a result of seed pre-treatments was found in the range of 3.43 ± 0.22 % (water boiled samples) to 32.18 ± 0.34 % (autoclaved samples). The oil samples at day 0 indicated the maximum color (R and Y Lovibond scale) value for oven drying while at storage day 60 (25 ± 2 °C), the highest color value was found for autoclave pre-treatment. The slightly increasing trend of color values for all treatments was observed during the storage period. The lowest iodine value (182.83 ± 1.18 g/100 g at storage day 0 & 173.49 ± 1.21 g/100 g at storage day 60, 25 ± 2 °C) was calculated for autoclaved samples while the maximum iodine value (193.42 ± 1.14 g/100 g at storage day 0 & 190.36 ± 1.17 g/100 g at storage day 60, 25 ± 2 °C) was recorded for raw chia samples. The significant increasing trend for all treatments was observed in case of peroxide value and free fatty acids production during storage. Maximum decrease in linoleic (35 %) and α-linolenic (18 %) fatty acids was observed in autoclaved samples. The oil from pre-treated seed samples obtained decreasing scores for sensory parameters throughout the storage period at different conditions.

**Conclusions:**

As a result, chia seeds are an important source of lipids and essential fatty acids. The water boiling and high temperature processing of chia seeds provides instability to lipids during storage at room temperature. However, detailed investigation is required on the processing performance and storage stability of food products supplemented with pre-treated chia seeds and furthers their effect on biological system.

## Background

Chia (*Salvia hispanica* L.) is a tropical and subtropical climates herbaceous plant from the mint family (*Lamiaceae*) which produces tiny, flavorless and white or dark brown seeds. Chia seeds have oval shape with approximately 1.9–2 mm long, 1–1.4 mm wide and 0.8–1 mm thickness diameter [1]. Commercial field production for chia seeds have been estimated 550–600 kg/ha and widely cultivated in Mexico, America, Canada, Chile, Australia, New Zealand and Southeast Asia for different purposes [2, 3]. Chia seed has in its composition 22–24 g/100 g protein, 26–41 g/100 g carbohydrates, 18–30 g/100 g dietary fiber, 4–6 g/100 g ash, vitamins, antioxidants, minerals, 91–93 g/100 g dry matter and 32–39 g/100 g oil contents [4]. The quality of edible oils is important for their acceptance as food or medicinal supplements because the fatty acids composition is a primary factor in its formulation. The lower content of saturated fatty acids (palmitic and stearic acids), adequate concentration of linoleic acids (18–20 %) and higher content of alpha-linolenic fatty acids (55–60 %) makes chia oil as a preferred and appealing choice for healthy food and cosmetic applications [5]. Human consumption of chia in diet is mainly from the extracted oil through its incorporation into different food formulations such as composite flours, confections, cookie snacks, salad dressing, cereal bars, fruit juices, breads, jellies, yoghurt and emulsions as well as health supplement for biological system [6]. Chia seeds and extracted oil contents have been safely utilize in animal feeds to increase the polyunsaturated fatty acids and decrease the cholesterol levels in meat and egg products [7]. Research studies have reported that seeds and biochemical components from *Salvia hispanica* L. helps to maintain serum lipid level, increase the satiety index, prevent from cardiovascular diseases, inflammation, nervous system disorders and diabetes [8, 9]. A larger supply of α-linolenic fatty acids improves the antioxidant status, the capacity of fat oxidation, n-3 long chain polyunsaturated fatty acids content and reduces the activity of fat synthesis biological tissue [10].

Various extraction methods are being utilized for oil extraction from chia seeds including solvent extraction, supercritical fluid and cold or hot pressing which differ in oil yield efficiency [4, 11]. On the other hand, both the oil yield and fatty acids composition of chia seed is affected by several factors, such as seed variety, pre-treatment method, seed storage conditions and size reduction practices. Long chain polyunsaturated fatty acids are highly susceptible to lipid oxidation which is considered as a serious problem that often leads to loss of shelf-life, consumer acceptability, functionality, nutritional value and safety. Consequently, the presence of fatty acid oxidation products in human foods, especially the aldehydes have ability to crosslink to proteins and bind covalently to nucleic acids and ultimately enhance aging, mutagenesis, and carcinogenesis mechanisms [12, 13]. To the best of our knowledge, however, the comparison impact of different chia seed pre-treatments on lipids stability during storage at different temperatures has not been reported previously in literature. From nutritional point of view, suitable pre-treatment techniques are required to produce high quality chia products in the food processing industries, to offer a healthy product for population and during clinical practices against different diseases. The aims of this investigation, therefore, were to study the effect of different seed pre-treatments such as particle size reduction, water boiling, microwave roasting, oven heating, and autoclaving on yield and sensory acceptability of chia seed oil at different storage intervals and conditions. Furthermore, the influence of these parameters on fatty acids composition and oxidative stability in terms of free fatty acid and peroxide values was also determined.

## Methods

### Raw materials and seed pre-treatments

The seeds of chia were procured from local supermarket, Faisalabad, Punjab, Pakistan. The unprocessed seeds were cleaned to remove any debris or field dirt and any other extraneous matters. The chia seeds were weight (100 ± 0.1 g) using the electronic weighing balance (Model Kern 440–35 N) for each treatment. The proximate composition of chia seeds was determined following previous methods [14]. Whole unprocessed chia seeds were milled through a China Grinder and passed through sieves to obtain different particle size fractions (coarse, seed particle size ≥ 10 mm; medium, seed particle size ≥ 5 mm; fine, seed particle size ≤ 5 mm). The water boiling pre-treatment included the water (500 mL) and chia seeds boiling together for 5 min in closed kettle. Microwave roasting was performed with a household convection microwave oven (Model RHM 2507) with 900 W output under the operating frequency of 2450 MHz for 2.5 min according to method described by Hongzhi et al. [15]. The oven drying of samples was carried out at 105 ± 5 °C for 1 h using the drying oven (Model 202-0A) with voltage 220 V, frequency 50 Hz and power 1.2 KW specifications. Autoclaving of chia seeds was completed in autoclave at 121 °C and 15 lbs for 15 min [16]. Chia seed oil for each treatment was obtained by pressing the seeds in low temperature Mini Oil Presser (Model 6YL-550; china made) with capacity 2–3 kg/h. The extracted oil samples were stored in Tin cans of 1-kg capacity (0.20 mm sheet thickness with flange of 2-mm) at 25 ± 2 °C and 4 ± 1 °C for 60–days, respectively.

### Physical characteristics, oxidative parameters and fatty acids composition of chia seed oil

The color (R and Y value) of chia oil samples was determined by Lovibond Tintometer using 5.25” quartzcell. Melting point of samples was assessed by AOAC [17] Method No. 920–157. The refractive index of samples was recorded by means of Abbe’s refractometer at 40 °C following the protocol No. Cc 7–25 as described in AOCS [18]. Iodine value of oil samples was analyzed by following the AOCS [18] Method No. (Cd 1d–92). 0.5 g of the oil sample, 25 mL chloroform, 25 mL of Wij’s solution, 20 mL of 10 % potassium iodide (KI) solution and 10 mL distilled water in 500 mL iodine flask was allowed to stand for 50 min in dark with shaking occasionally. Titrated the free iodine with standard 0.1 N sodium thiosulphate (Na_2_S_2_O_3_) solution in the presence of starch solution as indicator. Also conducted a blank determination without the sample. Iodine was calculated by using the formula as

$$ \frac{\left(\mathrm{V}\mathrm{o}\mathrm{l}.\ \mathrm{o}\mathrm{f}\ {\mathrm{Na}}_2{\mathrm{S}}_2{\mathrm{O}}_3\ \mathrm{used}\ \mathrm{f}\mathrm{o}\mathrm{r}\ \mathrm{blank}-\mathrm{V}\mathrm{o}\mathrm{l}.\ \mathrm{o}\mathrm{f}\ {\mathrm{Na}}_2{\mathrm{S}}_2{\mathrm{O}}_3\ \mathrm{used}\ \mathrm{f}\mathrm{o}\mathrm{r}\ \mathrm{sample}\right) \times \mathrm{Normality}\ \mathrm{o}\mathrm{f}\ {\mathrm{Na}}_2{\mathrm{S}}_2{\mathrm{O}}_3 \times 12.69}{\mathrm{Weight}\ \mathrm{o}\mathrm{f}\ \mathrm{the}\ \mathrm{o}\mathrm{il}\ \mathrm{taken}} $$

Peroxide value of oils samples was determined by AOCS [18] Method No. Cd 8–53. 2 g of oil sample, 30 mL of solution mixture (acetic acid and chloroform 3:2) and 0.5 mL of freshly prepared saturated KI solution was taken in a 250 mL iodine flask provided with well fitted glass stopper. Allowed the solution to stand for about 2 min with occasional shaking and added the 30 mL of distilled water. Titrated the liberated iodine against 0.1 N Na_2_S_2_O_3_ solution containing the 0.5 mL of starch solution as indicator. A blank reading was taken under the similar conditions at the same time. The peroxide value was calculated by using the relationship

$$ \frac{\left(\mathrm{V}\mathrm{o}\mathrm{l}.\ \mathrm{o}\mathrm{f}\ {\mathrm{Na}}_2{\mathrm{S}}_2{\mathrm{O}}_3\ \mathrm{used}\ \mathrm{f}\mathrm{o}\mathrm{r}\ \mathrm{blank}-\mathrm{V}\mathrm{o}\mathrm{l}.\ \mathrm{o}\mathrm{f}\ {\mathrm{Na}}_2{\mathrm{S}}_2{\mathrm{O}}_3\ \mathrm{used}\ \mathrm{f}\mathrm{o}\mathrm{r}\ \mathrm{sample}\right) \times \mathrm{Normality}\ \mathrm{o}\mathrm{f}\ {\mathrm{Na}}_2{\mathrm{S}}_2{\mathrm{O}}_3 \times 1000}{\mathrm{Weight}\ \mathrm{o}\mathrm{f}\ \mathrm{the}\ \mathrm{o}\mathrm{il}\ \mathrm{taken}} $$

Free fatty acid value of sample oils was analyzed by AOCS [18] Method No. Ca 5a–40. Briefly, take the 10 g of well mixed and entirely liquid oil in a 250 mL conical flask. Added the specified quantity of hot neutralized alcohol (50 mL) in it. Then, added the 2–3 drops of phenolphthalein (1 %) as indicator. Titrated the solution against the 0.1 N NaOH solution shaking vigorously to the appearance of first permanent pink color end point. Free fatty acid value was calculated as

$$ \frac{\mathrm{mL}\ \mathrm{of}\ \mathrm{NaOH}\ \mathrm{used} \times \mathrm{Normality}\ \mathrm{of}\ \mathrm{NaOH} \times 28.2}{\mathrm{Weight}\ \mathrm{of}\ \mathrm{sample}} $$

The fatty acids profile of extracted oil samples was determined by the method Ce 1f–96 given in AOCS [18]. The oil sample (50 μL) was methyated in the presence of 4 mL KOH (1 M) at room temperature for 1 h in order to convert fatty acids into their respective methyl esters. The resultant fatty acid methyl esters (FAMEs) were extracted with GC grade n–hexane and analyzed by Gas Chromatograph (Varian 3900) apparatus equipped with an auto sampler, flame–ionization detector (FID) and supelco wax column (30 m x 0.25 μm film coating). The samples (1 μL) were injected with Helium (1 mL/min) as a carrier gas onto the column, which was programmed for operating conditions such as column oven temperature 160 °C @ 0 min with subsequent increase of 3 °C/min until 180 °C. The column oven temperature was increased from 180 °C to 220 °C @ 1 °C/min and was held for 7.5 min at 220 °C. Split ratio was 50 % with injector 240 °C and detector 250 °C temperatures. The peak areas and total fatty acids composition were calculated for each sample by retention time using Varian Chem Station software. The standards of fatty acids methyl esters purchased from Sigma-Aldrich were also run under the same conditions for comparison with experimental samples.

### Sensory evaluation of chia seed oil

Twenty panel judges consisting of experienced and untrained panelists carried out the sensory analysis of pre-treated chia oil samples according to the instructions given by Meilgaard et al. [19]. Each judge gave written informed consent after explanation of risks and benefits of participation prior to the study. The panelists were provided informative instructions and brief definitions of attributes such as appearance, flavor and overall acceptability. Each panelist received the samples assigned with random three–digit code numbers. Each panelist was asked to list their preference on a 9–cm comparison line (1 = dislike extremely to 9 = like extremely). The sensory analysis was performed and completed at 0, 30 and 60 days of storage interval for experimental treatments.

### Statistical analysis

The data of oil yield obtained for each treatment was subjected to statistical analysis to determine the level of significance by using the software package (Statistic 8.1) according to the method described [20]. The average of the three runs was reported as the measured value with standard deviation. The Duncan’s multiple range (DMR) test was used to estimate the level of significance that existed between the mean values. The sample analysis for storage stability and consumer acceptability were carried out in triplicate and calculated the significant differences among means at a probability level of 5 %.

## Results and discussion

### Characterization of raw chia seed

The proximal composition of chia seeds consisted of 6.16 ± 0.24 % moisture, 34.84 ± 0.62 % oil, 18.21 ± 0.45 % protein, 4.16 ± 0.37 % ash, 23.12 ± 0.29 % fiber and 14.18 ± 0.23 % nitrogen contents. The chia seed presented a good source of protein (25.32 g/100 g), oil (30.22 g/100 g) and total dietary fiber (37.50 g/100 g) with predominant insoluble fiber (35.07 g/100 g) [21]. Seeds from three *Salvia* sources were ranged in concentration from 19.0 to 26.5 % for protein, 15.9 to 34.1 % for oil and 47.1 to 59.8 % for total dietary fiber contents [22]. Porras-Loaiza et al. [23] reported that the chia seeds had high contents of protein (18.5–22.3 %), fat (21.5–32.7 %) and fiber (20.1–36.1 %) while according to region, Mexican chia seed possessed the fat content ranged between 21.5 and 32.7 % and Ecuadorian chia seed fat content were between 27.5 and 32.8 %. Another study documented that the chia seeds are composed of protein (15–25 %), fats (30–33 %), carbohydrates (26–41 %), high dietary fiber (18–30 %), ash (4–5 %) and dry matter (90–93 %) [1]. The results reported in the present study are in accordance with other studies carried out in different countries [24–29].

### Effect of seed pre-treatments on chia oil yield

A major goal in chia oil production is to find an appropriate method to recover it from the seeds with preserving oil quality. The oil yield as a result of seed pre-treatment was found 29.72 ± 0.55 % (coarse), 17.34 ± 0.48 % (medium), 26.52 ± 0.36 % (fine), 3.43 ± 0.22 % (water boiled), 31.17 ± 0.29 % (microwave roasted), 23.32 ± 0.45 % (oven dried) and 32.18 ± 0.34 % (autoclaved) samples, respectively. In previous studies, the 0.113 g/g dry solids in chia seed and 30 °C processing temperature was observed the best processing combination to maximize oil yield [30]. An extraction temperature of 50 °C, a solvent to seed ratio of 12 mL/g and 40 min of extraction were identified as the ultrasound optimal conditions with 27.24 % chia oil yield [31]. The oil yield documented here is in the same range 20.3 to 33.6 % reported by Ixtaina et al. [4] and Vargas et al. [32] but was found lower than data published by Martínez et al. [30] and Ayerza [33]. This difference in oil yield could be connected to the difference in climatic conditions, agronomic practices, fertilization regimes and irrigation practices. The lowest oil yield in case of water boiling process could be attributed to high seed moisture contents which may resulted in poor oil recoveries because of insufficient friction during Mini oil pressing. Another reason is the physical formation of an external gelatinous structure on seed meal with water-holding properties. These observations are consistent with the fact that seed hydration affects the mucilage structure characteristic of chia [30, 34, 35]. Therefore, the optimal moisture content should be determined before pressing the chia seed for oil extraction process [34, 36, 37]. On the other hand, the decrease of seed moisture contents during thermal processing resulted in an increase of oil yield and the highest values of oil yield were obtained with the lowest values of seed moisture contents [30].

### Effect of seed pre-treatments on physical parameters of chia oil

**Fig. 1:**
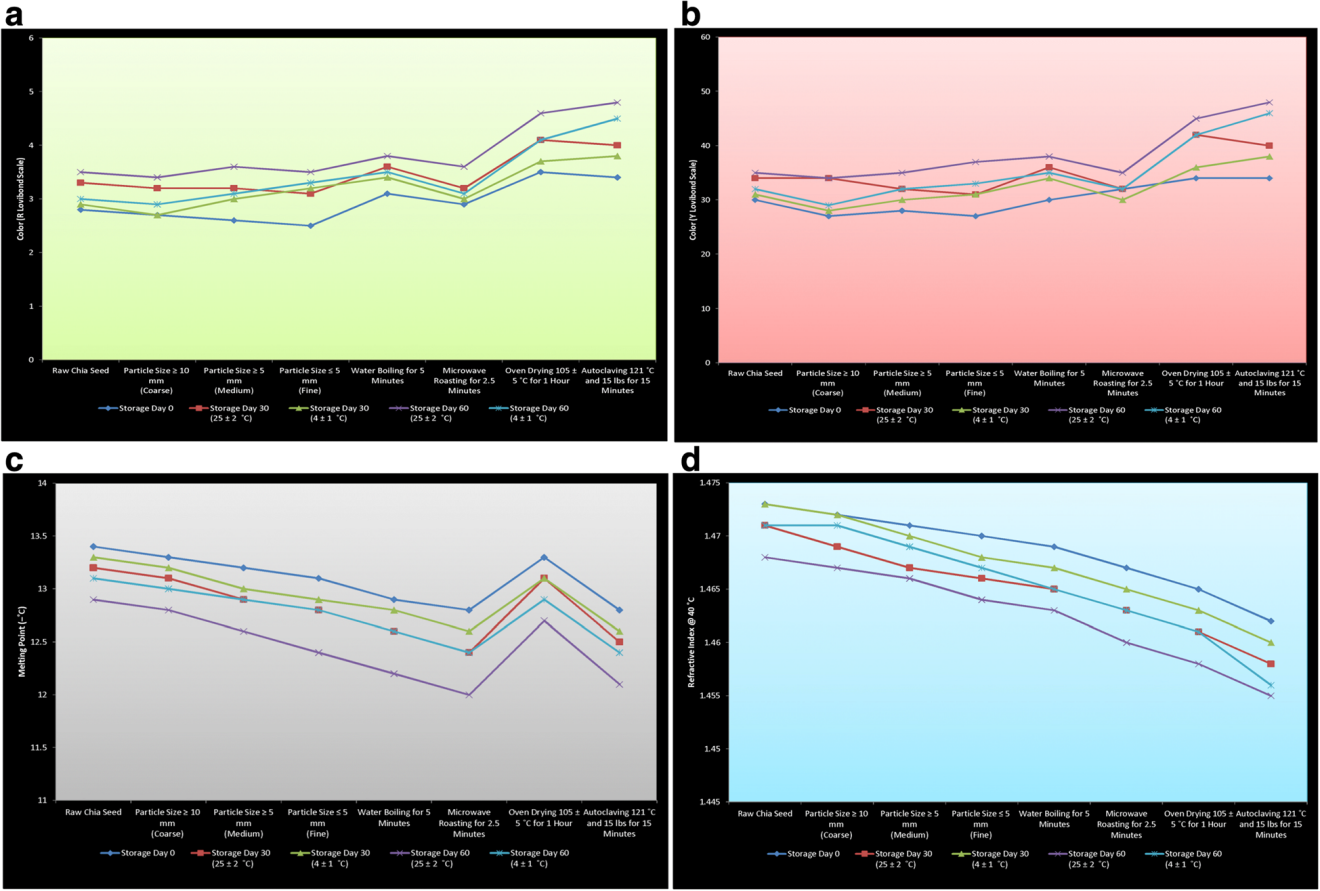
Effect of seed pre-treatment processing techniques on physical parameters (**a** Color (R Lovibond Scale), **b** Color (Y Lovibond Scale), **c** Melting Point and **d** Refractive Index) of chia seed oil at different storage intervals

The effect of seed pre-treatment processing techniques on physical parameters (color, melting point and refractive index) of chia seed oil at different storage intervals have been presented in Fig. 1. There are no color standards for chia seed oils and therefore the Lovibond color R and Y measurements could be used for color classification. The results regarding the color (R and Y Lovibond scale) at day 0 indicate the maximum value for oven drying pre-treatment while at storage day 60 (25 ± 2 °C), the highest value was found for autoclaving pre-treatment (Fig. 1a & b). However, the slightly increasing trend of color values for all treatments was observed during the storage period. The significant differences were found between the L* and a* values of the CIELAB chia oil [4]. In the same study from authors, the lowest L* values found in oils obtained by pressing were appeared consequently darker which is also supported by the results mentioned by Melendez-Martınez et al. [38]. It is noteworthy to mention that the color of vegetable oils is associated with the total pigment content and the presence of carotenoids in chia seed oils [4]. The melting point of pre-treated samples (Fig. 1c) showed that the oil extracted from raw chia seed had lowest melting point (–13.4 °C) while highest melting point (–12.8 °C) was noted for autoclaved samples. The increasing trend in melting point for all treatments was observed during the storage life. In a similar way, the decreasing trend for refractive index of oil extracted from different pre-treated seeds was seen at different storage intervals (Fig. 1d). Refractive index ranged from 1.4763 to 1.4798 recording significant differences between the oils obtained by different extraction systems [4].

### Effect of seed pre-treatments on oxidative parameters of chia oil

Table 1 shows the effect of seed pre-treatment processing techniques on oxidative stability of chia seed oil at different storage intervals. The lowest iodine value (182.83 ± 1.18 g/100 g at storage day 0 & 173.49 ± 1.21 g/100 g at storage day 60, 25 ± 2 °C) was calculated for autoclaved samples while the maximum iodine value (193.42 ± 1.14 g/100 g at storage day 0 & 190.36 ± 1.17 g/100 g at storage day 60, 25 ± 2 °C) was recorded for raw chia samples. Slightly differences were found between the iodine values of other treatments at the different conditions studied. The high iodine value is related to the fatty acid composition of chia oil rich mainly in polyunsaturated fatty acids. Ixtaina et al. [4] found somewhat higher iodine values (208.5–215.0) when compared with the present study results. Peroxide value and free fatty acids contents were found low which indicates the high quality of the raw chia samples. The peroxide value parameter for raw chia oil varied between 0.69 and 2.67 (meq/kg oil) [30]. The raw chia oil presented a low peroxide index value (2.56 meq peroxide/kg) [21]. Ayerza and Coates [26] reported the peroxide value (2.7–3.8 meq oxygen/kg) in chia oil from South America origin while Ixtaina et al. [39] described the peroxide value (1.0 meq peroxide/kg) in chia oil from Argentina origin. Furthermore, the chia oil samples present the lower peroxide values than other vegetable oils like flaxseed oil [40]. The increasing trend for all treatments was observed significantly (*p* ≤ 0.05) in case of peroxide value and free fatty acids production during storage. It seems true that the increase of seed moisture in water boiling treatment affected negatively the peroxide index and samples stored at 25 ± 2 °C after 60 days were found unacceptable as peroxide value (10.69 ± 0.22 meq peroxide/kg) exceeds the maximum permissible limit (10 meq peroxide/kg) [41], however, the chemical quality of oils obtained in all other treatments was acceptable and in the permissible limit. Similar trend of unacceptance of samples was found for autoclaved pre-treated chia seeds after storage life at room temperature. The peroxide value of 10 meq/kg was observed for chia oils stored at 4 °C while values greater than 10 meq/kg were observed between 60 and 120 days when stored at 20 °C [39]. Similar results were reported by Ayerza and Coates [42]. Values higher than 10.0 meq O_2_/kg were observed between 120–240 days for sunflower and chia oil blends stored at 20 ± 2 °C [43]. As expected, it is indicated from the Table 1 that oil samples at day 0 had lower free fatty acids than the oils stored at 4 ± 1 °C and 25 ± 2 °C after 60 days. High temperature and water contents had a strong influence on oil oxidation. Moreover, the free fatty acids of oils stored at 4 ± 1 °C recorded lower values than the oils stored at 25 ± 2 °C indicating the relatively less occurrence of oxidation at low temperature. None of the samples exceeded the upper limit of free fatty acids established by the Codex Alimentarius [41] except the water boiled and autoclaved samples after 60 days of storage at 25 ± 2 °C. The chia oil samples possessed the free fatty acids in the range of 1.3 ± 0.1 on % oleic acid basis [4]. The average free fatty acid values ranged between 0.70 and 2.05 mg KOH/g oil and chia oils presented significantly higher free fatty acid values in solvent than in pressure system [4]. Raw chia oil possessed the bioactive components (tocopherols, polyphenols, myricetin, quercetin, kaempferol, chlorogenic acid and 3,4-dihydroxyphenylethanol-elenolicacid dialdehyde and carotenoids) which may be responsible for keeping low peroxide values and consequently may present a good oxidative stability [4, 21, 43]. Deterioration occurs through rancidity resulting from oxidation which takes place at the double bond sites in the triacyleglycerol molecules. It is evident from earlier studies that the oxidation process causes great economic loses to the food industry and allied consumers.

**Table 1:** Effect of seed pre-treatment processing techniques on oxidative stability of chia seed oil at different storage intervals

Analyzed Parameter	Treatment							
	Raw Chia Seed	Particle Size ≥ 10 mm (Coarse)	Particle Size ≥ 5 mm (Medium)	Particle Size ≤ 5 mm (Fine)	Water Boiling for 5 min	Microwave Roasting for 2.5 min	Oven Drying 105 ± 5 °C for 1 h	Autoclaving 121 °C and 15 lbs for 15 min
Iodine Value (g/100 g)								
Storage Day 0	193.42 ± 1.14^a^	193.10 ± 1.19^a^	192.92 ± 1.23^a^	192.81 ± 1.26^a^	190.73 ± 1.35^a^	189.74 ± 1.25^a^	185.90 ± 1.22^a^	182.83 ± 1.18^a^
Storage Day 30 (25 ± 2 °C)	189.93 ± 1.12^b^	190.72 ± 1.18^b^	190.54 ± 1.14^b^	190.33 ± 1.17^b^	185.65 ± 1.22^c^	184.86 ± 1.12^b^	181.12 ± 1.25^c^	177.15 ± 1.26^c^
Storage Day 30 (4 ± 1 °C)	192.24 ± 1.23^a^	192.74 ± 1.13^a^	192.66 ± 1.18^a^	192.45 ± 1.12^a^	188.97 ± 1.15^b^	188.38 ± 1.15^a^	184.24 ± 1.14^a^	180.67 ± 1.19^b^
Storage Day 60 (25 ± 2 °C)	185.65 ± 1.34^c^	186.86 ± 1.16^c^	186.78 ± 1.25^c^	186.37 ± 1.26^c^	181.29 ± 1.21^d^	180.70 ± 1.21^c^	178.36 ± 1.32^d^	173.49 ± 1.21^d^
Storage Day 60 (4 ± 1 °C)	190.36 ± 1.17^b^	190.68 ± 1.25^b^	190.50 ± 1.21^b^	190.49 ± 1.23^b^	186.31 ± 1.23^c^	185.52 ± 1.33^b^	183.18 ± 1.22^b^	177.51 ± 1.28^c^
Peroxide Value (meq *peroxide*/kg)								
Storage Day 0	1.80 ± 0.16^d^	1.76 ± 0.10^e^	1.82 ± 0.18^d^	1.84 ± 0.14^d^	1.62 ± 0.14^e^	1.92 ± 0.13^d^	0.46 ± 0.10^d^	1.96 ± 0.10^e^
Storage Day 30 (25 ± 2 °C)	3.92 ± 0.22^b^	4.18 ± 0.13^b^	4.24 ± 0.20^b^	4.36 ± 0.17^b^	5.45 ± 0.16^c^	4.39 ± 0.15^b^	2.37 ± 0.12^b^	5.71 ± 0.11^c^
Storage Day 30 (4 ± 1 °C)	2.44 ± 0.12^c^	2.60 ± 0.15^d^	2.76 ± 0.21^c^	2.88 ± 0.19^c^	3.13 ± 1.17^d^	3.19 ± 0.16^c^	1.52 ± 1.13^c^	3.42 ± 0.15^d^
Storage Day 60 (25 ± 2 °C)	5.66 ± 0.17^a^	5.72 ± 0.18^a^	5.88 ± 0.15^a^	5.90 ± 0.20^a^	10.69 ± 0.22^a^	7.85 ± 0.18^a^	3.61 ± 1.14^a^	11.27 ± 0.16^a^
Storage Day 60 (4 ± 1 °C)	3.58 ± 0.19^b^	3.64 ± 0.22^b^	3.70 ± 0.17^b^	3.82 ± 0.22^b^	7.48 ± 0.24^b^	5.73 ± 0.14^b^	2.49 ± 0.15^b^	7.95 ± 0.18^b^
Free Fatty Acids (% oleic acid)								
Storage Day 0	1.07 ± 0.13^d^	1.10 ± 0.16^d^	1.12 ± 0.12^e^	1.15 ± 0.10^e^	1.02 ± 0.14^e^	1.13 ± 0.11^e^	0.62 ± 0.10^e^	1.14 ± 0.13^e^
Storage Day 30 (25 ± 2 °C)	1.71 ± 0.16^b^	1.78 ± 0.19^b^	1.81 ± 0.11^c^	1.83 ± 0.13^c^	2.31 ± 0.17^c^	2.14 ± 0.14^b^	1.84 ± 0.11^b^	2.67 ± 0.16^c^
Storage Day 30 (4 ± 1 °C)	1.32 ± 0.15^c^	1.37 ± 0.14^c^	1.40 ± 0.13^d^	1.44 ± 0.17^d^	1.69 ± 0.18^d^	1.34 ± 0.19^d^	0.98 ± 0.13^d^	1.86 ± 0.19^d^
Storage Day 60 (25 ± 2 °C)	2.23 ± 0.18^a^	2.28 ± 0.15^a^	2.32 ± 0.14^a^	2.37 ± 0.15^a^	5.22 ± 0.19^a^	3.88 ± 0.15^a^	2.35 ± 0.12^a^	5.56 ± 0.22a
Storage Day 60 (4 ± 1 °C)	1.65 ± 0.17^b^	1.69 ± 0.11^b^	2.03 ± 0.15^b^	2.16 ± 0.18^b^	3.15 ± 0.21^b^	1.82 ± 0.12^c^	1.16 ± 0.14^c^	4.06 ± 0.21^b^

*Values represent the mean* ± *standard deviation*; *n* = *3*

^*a*,*b*,*c*,*d*,*e*^ *Means in a column with different superscripts for individual analyzed parameter were significantly different* (*p* ≤ *0.05*)

Oxidative modification of lipids has long been regarded as a deleterious process responsible for significant changes in the chemical properties of the molecules, loss of function and generation of cytotoxic and genotoxic compounds especially oxidized lipids-derived aldehydes and peroxides. Such lipid peroxidation products have much more stable state and therefore can easily diffuse from their site of generation to remote locations for damage to biological tissues [44, 45]. The repeated consumption of such oxidized fats in the diet poses a chronic threat to human health by selective alterations in cell signaling, protein, DNA damage and dysfunction of organs such as liver, kidney, lung and the gut [46]. However, the oxidative stability of lipid fraction in complex food system is dependent on the composition, concentrations of reaction substrates, prooxidants and antioxidants. Therefore, decreasing the formation of lipid peroxidation products or scavenging them chemically could be beneficial in limiting the deleterious effects of reactive oxygen species in various pathological conditions. This could be potentially achieved by enhancing the endogenous oxidation control systems of foods through dietary supplementation of antioxidants [4, 47].

### Effect of seed pre-treatments on fatty acids composition of chia oil

The knowledge of the triacylglycerol profile of oil is useful to direct its proper use by the chemical, food and pharmaceutical industries [48] and also the current concern for fat intake in the world has raised the question of the individual fatty acid impact on health. The raw materials, chia seed oil from different pre-treated samples, were analyzed for their fatty acids composition. Chia seed possessed appreciable amounts of ω-3 alpha-linolenic acid and ω-6 linoleic acids. The palmitic (6.76 ± 0.15 %), oleic (8.34 ± 0.19 %), linoleic (12.14 ± 0.22 %) and α-linolenic (60.56 ± 1.22 %) fatty acids were predominantly present in raw chia oil at storage day 0 (Table 2). The quality and composition of fatty acids constituents of chia oils were influenced by the pre-treatment process. Maximum palmitic, stearic and oleic fatty acids concentration was noted in autoclaved samples at storage day 60 (25 ± 2 °C). The linoleic and α-linolenic acid contents are also considered as an indicator of chia seeds suitability for incorporation in healthy foods for discerning consumers. At day zero, the raw and processed chia seeds presented the highest values of linoleic and α-linolenic acids. These values tended to slightly decrease during the storage period. It seems that raw chia seeds were less influenced by storage time, showing a less marked decreasing trend over time. During storage, a marked decrease in linoleic acid content was observed for processed chia samples. The maximum decrease in amount of linoleic (35 %) and α-linolenic (18 %) fatty acids was observed in autoclaved samples. There is little information about chia seed oil and the influence of the extraction system on its fatty acid profile and physico-chemical characteristics [32]. Martínez et al. [30] found the abundance order of fatty acids in chia oil as α-linolenic (C_18:3_); linoleic (C_18:2_); oleic (C_18:1_); palmitic (C_16:0_) and stearic acid (C_18:0_). de Mello et al. [31] also reported the linolenic (∼66 %) and linoleic acid (∼20 %) as the main fatty acid constituents of ultrasound treated chia seed oil. The final extract of chia oil obtained by SC-CO_2_ under different conditions and Soxhlet extraction contained mainly alpha-linolenic (64.9–65.6 %), linoleic (19.8–20.3 %), palmitic (6.2–6.7 %), oleic (5.0–5.5 %) and stearic acid (2.7–3 %) [4]. Another study reports that the chia seed oil contains low percentage of saturated fatty acids and abundant in polyunsaturated fatty acids such as linoleic (19.1 ± 0.8 %) and linolenic (64.7 ± 0.8 %) acids [39]. On an average it contains about 64 % ω-3 and 19 % ω-6 fatty acids [11]. The main fatty acids in chia seed oil, ranked order of abundance, were α-linolenic acid > linoleic acid > palmitic acid ∼ oleic acid > stearic acid [21]. These findings are in agreement with those reported in present study and mentioned in earlier studies [4, 25, 26, 29, 33, 40, 49, 50]. Ecuadorian seeds (linolenic acid 63.3–67.3 %) have slightly higher content than that found in seeds from Michoacan, Oaxaca, Chiapas and Puebla (59.9–63.4 %) [23]. Gas chromatography analysis of the oil composition showed the presence of palmitic, stearic, oleic, linoleic and a-linolenic fatty acids in both white and black-spotted color seeds from all locations whereas the larger differences found in oil content and fatty acid composition was due to location (because of the environmental differences) rather than chia seed coat color [51]. It has been found that the lipids are rich in linolenic acid which accounted for 58.2 % in flax, 60.9 % in perilla and 59.8 % in chia [50]. The alteration in fatty acids composition during thermal treatment of raw materials may be due to lipolytic activity, interactions between lipids and other constituents or processing conditions. The present study shows that storage and heat partially decrease the amount of polyunsaturated fatty acids content. Therefore this must be taken into consideration when selecting the operating temperature and storage conditions [52]. Seed size reduction and slight heat treatment practice is an interesting methodology because it is possible to achieve a chia oil yield close to that obtained from raw chia oil with a similar fatty acid composition using an environmentally friendly process.

**Table 2:** Effect of seed pre-treatment processing techniques on fatty acids composition of chia seed oil at different storage intervals

Analyzed Parameter	Treatment							
	Raw Chia Seed	Particle Size ≥ 10 mm (Coarse)	Particle Size ≥ 5 mm (Medium)	Particle Size ≤ 5 mm (Fine)	Water Boiling for 5 Min	Microwave Roasting for 2.5 Min	Oven Drying 105 ± 5 °C for 1 h	Autoclaving 121 °C and 15 lbs for 15 min
C16:0 (Palmitic acid)								
Storage Day 0	6.76 ± 0.15^c^	6.85 ± 0.16^c^	6.92 ± 0.11^c^	6.95 ± 0.10^c^	7.07 ± 0.13^d^	7.11 ± 0.11^d^	7.23 ± 0.19^d^	7.32 ± 0.18^d^
Storage Day 30 (25 ± 2 °C)	7.45 ± 0.13^b^	7.67 ± 0.18^b^	7.79 ± 0.13^b^	7.88 ± 0.12^b^	8.12 ± 0.15^b^	8.22 ± 0.12^b^	8.28 ± 0.17^b^	8.52 ± 0.19^b^
Storage Day 30 (4 ± 1 °C)	6.94 ± 0.14^c^	7.05 ± 0.20^c^	7.15 ± 0.15^c^	7.23 ± 0.14^c^	7.46 ± 0.16^c^	7.53 ± 0.13^c^	7.68 ± 0.18^c^	7.79 ± 0.14^c^
Storage Day 60 (25 ± 2 °C)	8.76 ± 0.18^a^	8.82 ± 0.14^a^	8.95 ± 0.17^a^	9.09 ± 0.16^a^	9.32 ± 0.17^a^	9.41 ± 0.14^a^	9.65 ± 0.16^a^	9.81 ± 0.13^a^
Storage Day 60 (4 ± 1 °C)	7.54 ± 0.17^b^	7.57 ± 0.12^b^	7.71 ± 0.19^b^	7.87 ± 0.18^b^	8.04 ± 0.19^b^	8.12 ± 0.16^b^	8.23 ± 0.15^b^	8.41 ± 0.11^b^
C18:0 (Stearic acid)								
Storage Day 0	2.78 ± 0.07^c^	2.85 ± 0.08^c^	2.91 ± 0.09^c^	2.98 ± 0.10^c^	3.11 ± 0.06^d^	3.24 ± 0.11^d^	3.31 ± 0.09^d^	3.44 ± 0.11^d^
Storage Day 30 (25 ± 2 °C)	3.54 ± 0.09^b^	3.61 ± 0.10^b^	3.76 ± 0.11^b^	3.87 ± 0.11^b^	3.98 ± 0.07^b^	4.16 ± 0.12^b^	4.24 ± 0.08^b^	4.31 ± 0.09^b^
Storage Day 30 (4 ± 1 °C)	2.95 ± 0.10^c^	2.99 ± 0.11^c^	3.14 ± 0.12^c^	3.28 ± 0.13^c^	3.45 ± 0.09^c^	3.67 ± 0.14^c^	3.81 ± 0.07^c^	3.98 ± 0.11^c^
Storage Day 60 (25 ± 2 °C)	4.26 ± 0.11^a^	4.33 ± 0.13^a^	4.56 ± 0.14^a^	4.82 ± 0.12^a^	5.04 ± 0.08^a^	5.15 ± 0.15^a^	5.30 ± 0.06^a^	5.51 ± 0.12^a^
Storage Day 60 (4 ± 1 °C)	3.36 ± 0.12^b^	3.47 ± 0.14^b^	3.58 ± 0.13^b^	3.74 ± 0.11^b^	3.92 ± 0.10^b^	4.10 ± 0.17^b^	4.23 ± 0.11^b^	4.35 ± 0.13^b^
C18:1 (Oleic acid ω-9)								
Storage Day 0	8.34 ± 0.19^d^	8.56 ± 0.16^d^	8.62 ± 0.15^d^	8.69 ± 0.13^d^	8.89 ± 0.11^d^	9.05 ± 0.14^d^	9.26 ± 0.16^d^	9.38 ± 0.21^d^
Storage Day 30 (25 ± 2 °C)	9.12 ± 0.16^b^	9.23 ± 0.15^b^	9.36 ± 0.13^b^	9.49 ± 0.14^b^	9.73 ± 0.13^b^	9.89 ± 0.15^b^	10.05 ± 0.19^b^	10.23 ± 0.19^b^
Storage Day 30 (4 ± 1 °C)	8.72 ± 0.15^c^	8.84 ± 0.14^c^	8.98 ± 0.11^c^	9.16 ± 0.16^c^	9.27 ± 0.12^c^	9.43 ± 0.17^c^	9.56 ± 0.17^c^	9.78 ± 0.17^c^
Storage Day 60 (25 ± 2 °C)	10.45 ± 0.14^a^	10.66 ± 0.18^a^	10.88 ± 0.12^a^	11.37 ± 0.15^a^	11.52 ± 0.14^a^	11.69 ± 0.15^a^	11.75 ± 0.18^a^	11.92 ± 0.16^a^
Storage Day 60 (4 ± 1 °C)	9.09 ± 0.13^b^	9.20 ± 0.16^b^	9.42 ± 0.14^b^	9.73 ± 0.12^b^	9.86 ± 0.15^b^	9.94 ± 0.19^b^	10.16 ± 0.19^b^	10.38 ± 0.15^b^
C18:2 (Linoleic acid ω-6)								
Storage Day 0	12.14 ± 0.22^a^	12.03 ± 0.26^a^	11.94 ± 0.22^a^	11.81 ± 0.24^a^	11.79 ± 0.32^a^	11.62 ± 0.27^a^	11.45 ± 0.22^a^	11.28 ± 0.29^a^
Storage Day 30 (25 ± 2 °C)	9.37 ± 0.24^c^	9.25 ± 0.32^c^	9.09 ± 0.23^c^	8.93 ± 0.26^c^	8.76 ± 0.34^c^	8.54 ± 0.22^c^	8.32 ± 0.25^c^	8.13 ± 0.31^c^
Storage Day 30 (4 ± 1 °C)	10.44 ± 0.20^b^	10.29 ± 0.30^b^	10.03 ± 0.25^b^	9.86 ± 0.28^b^	9.67 ± 0.26^b^	9.65 ± 0.25^b^	9.42 ± 0.32^b^	9.15 ± 0.34^b^
Storage Day 60 (25 ± 2 °C)	8.66 ± 0.23^d^	8.55 ± 0.27^d^	8.13 ± 0.29^d^	8.01 ± 0.31^d^	7.91 ± 0.27^d^	7.83 ± 0.20^d^	7.45 ± 0.36^d^	7.26 ± 0.30^d^
Storage Day 60 (4 ± 1 °C)	9.46 ± 0.25^c^	9.31 ± 0.28^c^	9.16 ± 0.24^c^	9.04 ± 0.33^c^	8.84 ± 0.29^c^	8.68 ± 0.22^c^	8.36 ± 0.23^c^	8.03 ± 0.25^c^
C18:3 (α-Linolenic acid ω-3)								
Storage Day 0	60.56 ± 1.22^a^	60.43 ± 1.12^a^	60.14 ± 1.20^a^	59.99 ± 1.12^a^	59.73 ± 1.17^a^	59.45 ± 1.53^a^	59.26 ± 1.34^a^	58.84 ± 1.39^a^
Storage Day 30 (25 ± 2 °C)	55.42 ± 1.34^c^	55.13 ± 1.21^c^	54.92 ± 1.15^c^	54.43 ± 1.15^c^	54.24 ± 1.19^c^	53.88 ± 1.36^c^	53.41 ± 1.45^c^	52.61 ± 1.27^c^
Storage Day 30 (4 ± 1 °C)	58.6 ± 1.36^b^	58.24 ± 1.25^b^	57.98 ± 1.17^b^	57.19 ± 1.25^b^	56.4 ± 1.21^b^	55.60 ± 1.42^b^	54.82 ± 1.37^b^	53.42 ± 1.35^b^
Storage Day 60 (25 ± 2 °C)	52.7 ± 1.28^d^	52.34 ± 1.29^d^	51.93 ± 1.24^d^	51.48 ± 1.22^d^	50.87 ± 1.25^e^	49.94 ± 1.26^e^	49.22 ± 1.39^e^	48.52 ± 1.44^e^
Storage Day 60 (4 ± 1 °C)	56.3 ± 1.14^c^	55.96 ± 1.33^c^	55.19 ± 1.27^c^	54.81 ± 1.28^c^	53.36 ± 1.28^d^	52.76 ± 1.48^d^	52.14 ± 1.41^d^	51.63 ± 1.48^d^

*Values represent the mean* ± *standard deviation*; *n* = *3*

^*a*,*b*,*c*,*d*,*e*^ *Means in a column with different superscripts for individual analyzed parameter were significantly different* (*p* ≤ *0.05*)

### Effect of seed pre-treatments on sensory acceptance of chia oil

**Fig. 2:**
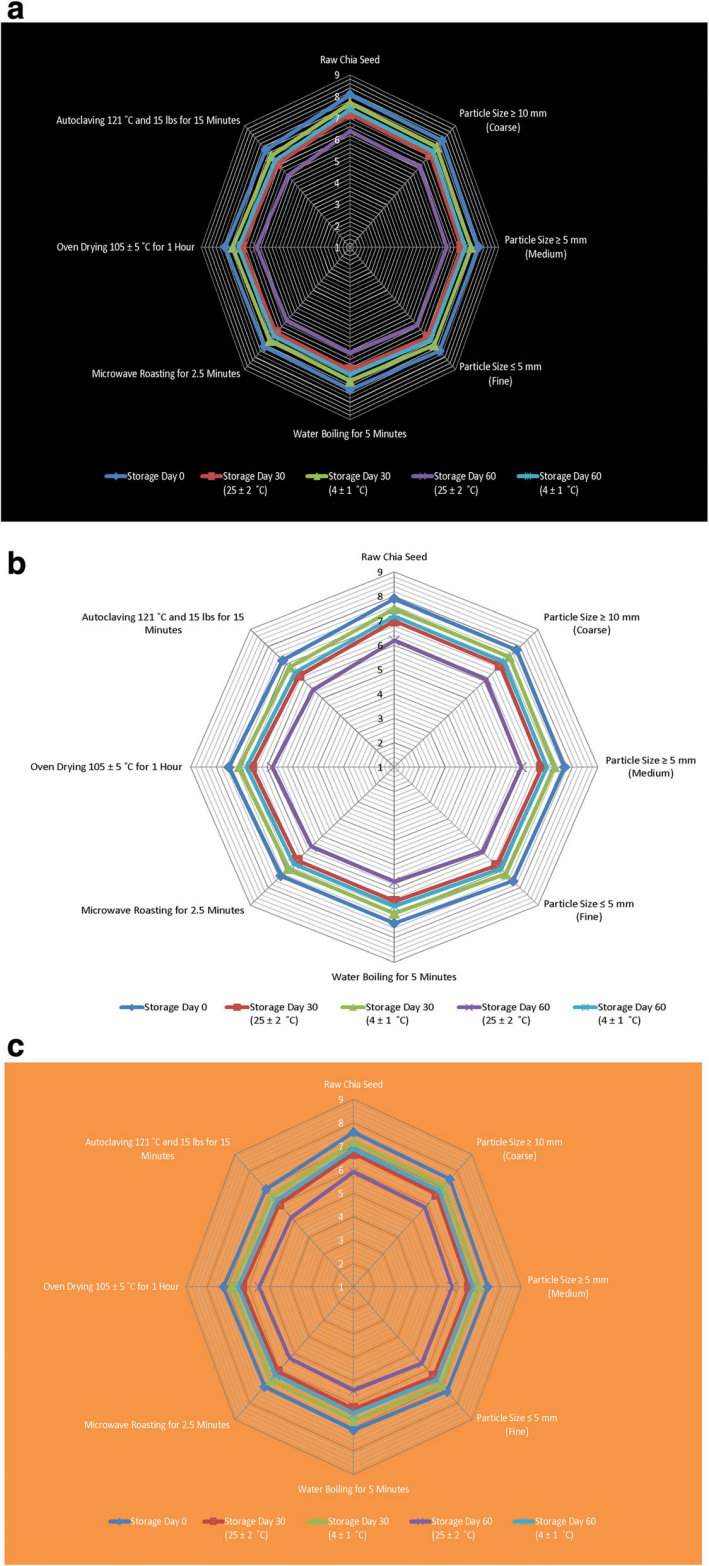
Effect of seed pre-treatment processing techniques on sensory evaluation (**a** Appearance, **b** Flavor and **c** Overall Acceptability) of chia seed oil at different storage intervals

Figure 2 shows the profile of the parameters as appearance, flavor and overall acceptability of oil samples extracted from pre-treated chia seeds stored at different intervals. The oil from all pre-treated seed samples obtained decreasing scores for sensory parameters throughout the storage period at different conditions. The organoleptic characteristics (light yellow oily liquid with peculiar odor) of raw chia oil grown in Cuba were similar to those obtained in other parts of the world [53]. The results in Fig. 2 indicate that the water addition, boiling and heat treatment negatively affected the preservation of appearance and flavor of the chia oil samples. The oxidation of polyunsaturated fatty acids in chia oil results in the generation of volatile compounds which many have unpleasant odors and are responsible for the off-flavors in the food products [39]. Polyunsaturated fatty acids oxidation generates volatile compounds that impart undesirable aromas and lead towards compromising the nutritional quality of the oil with limited shelf life [43]. Oxidation is considered as one of the most common causes of flavor quality deterioration for oils and oil products during storage period which accounts the great economic loses to the food industry and allied consumers.

## Conclusions

The results of the present study conclude that chia seeds are a good source of lipids and omega-3 fatty acids. The high content of polyunsaturated fatty acids makes chia seed oil very instable. Furthermore, the water boiling and autoclaving processing of chia seeds reduces the concentration of essential fatty acids during storage at room temperature. The innovative technologies to protect omega-3 polyunsaturated fatty acids using antioxidants, adequate preparation, refining and/or packaging of the oil extracted from pre-treated chia seeds, are needed. Industrially, further research should be conducted which would utilize the pre-treated chia seeds for the development of functional foods, or medicinal, pharmaceutical and other non-food industrial applications.
